# Systematic Literature Review on Indicators Use in Safety Management Practices among Utility Industries

**DOI:** 10.3390/ijerph19106198

**Published:** 2022-05-19

**Authors:** Mohamad Xazaquan Mansor Ali, Kadir Arifin, Azlan Abas, Mohd Akhir Ahmad, Muhammad Khairil, Muhammad Basir Cyio, Muhammad Ahsan Samad, Ilyas Lampe, Mahfudz Mahfudz, Muhammad Nur Ali

**Affiliations:** 1 Centre for Research in Development, Social and Environment (SEEDS), Faculty of Social Sciences and Humanities, Universiti Kebangsaan Malaysia (UKM), Bangi 43650, Malaysia; xazaquan1984@gmail.com (M.X.M.A.); azlanabas@ukm.edu.my (A.A.); mohdakhirahmad1982@gmail.com (M.A.A.); 2 Department of Occupational Safety and Health Malaysia, Ministry of Human Resources, Government Administrative Centre, Putrajaya 62530, Malaysia; 3 Faculty of Social and Political Sciences, Universitas Tadulako, Palu 94118, Indonesia; muh.khairil02@gmail.com (M.K.); ahsansamademail@gmail.com (M.A.S.); ilyaslampe7@gmail.com (I.L.); 4 Faculty of Agriculture, Universitas Tadulako, Palu 94118, Indonesia; basircyio@yahoo.com (M.B.C.); mahfudz62@gmail.com (M.M.); ali.mnur@yahoo.com (M.N.A.)

**Keywords:** safety management practices, leading indicators, safety performance, lagging indicators, occupational safety and health

## Abstract

Background: Workers in utility industries are exposed to occupational accidents due to inadequate safety management systems. Accordingly, it is necessary to characterize and compare the available literature on indicators used in safety management practices in the utility industries. Methods: The systematic literature review was based on the Preferred Reporting Items for Systematic Reviews and Meta-analysis statement. This study considered 25 related studies from Web of Science and Scopus databases. Results: Further review of these articles resulted in three mains performance indicators; namely, driven leading indicators, observant leading indicators, and lagging indicators consisting of 15 sub-indicators. Conclusions: Future studies should consider researching a more comprehensive range of utility industries, measuring subjective and objective indicators, integrating risk management into safety management practices, and validating the influence of leading indicators on safety outcomes. Further, researchers recommend including accidents, fatalities, lost time injuries, and near misses in safety outcomes.

## 1. Introduction

The International Labour Organization (ILO) estimates 340 million workplace accidents and 160 million people suffer from work-related illnesses worldwide every year, due to inadequate working conditions, leading to about 6000 workers dying every day [1]. One of the industries contributing to this statistic is the utility industry, which consists of water, electricity, and gas utilities that provide essential services to commodity providers and other industries, contributing to economic and social growth. Workers in the utility industry are subject to risks associated with their work activities and surroundings. In 2018, 405 fatal cases and 101,393 non-fatal cases of accidents were recorded by the ILO in the utility industry globally [1]. As a result, occupational accidents cause a burden on the injured individual and society, including monetary costs, such as wages lost and medical expenses, potential lifelong disability, and reduced quality of life [2]. Even with various programmes implemented by government authorities and organizations at the national level, the number of accidents at work is still high [3]. Thus, the effort implemented to control unsafe actions and conditions in the workplace is insufficient [4]. Accidents can be avoided by ensuring the safety level implemented in the organization is maintained and improved from time to time, through measuring indicators that proactively affect safety performance.

Safety performance is conventionally monitored by lagging indicators, such as accident rates, fatal accident rates, and dangerous occurrences, even though failure-focused control measures are less effective in driving continuous improvement efforts [5,6,7]. The lagging indicators method measures failures compared to current safety conditions and should not be considered a direct measurement of the level of safety in a working system [8] because incidents are rare occurrences with a low probability, making the accident frequency statistically unreliable due to variance restrictions [9]. The rare occurrence of incidents does not mean that the workplace is safer than other places where accidents occur, and it is not a clear performance indicator for hazard or risk management [10,11]. Therefore, lagging indicators that measure weakness rather than safety and ignore the different exposures of risks inherent in work activities should not be considered a direct measure of safety level in a working system [8,12,13,14,15]. Recent research is more focused on proactive action by measuring safety levels through OSH activities that bring safety management systems up to date towards the desired safety goals, enabling organizations to anticipate safety issues and potentially reduce OSH incidents [8,16,17,18,19].

Safety management is frequently considered a sub-system in overall organizational management and is implemented through many forms of safety management practices, the mechanism incorporated into an organization to control hazards at work [20,21]. Safety management consists of procedure, planning, information management, and supervision, which play significant roles in reducing occupational accidents [22]. On the other hand, the lack of a safety management system can lead to workplace accidents, among the most common causes of industrial disasters, such as the Bhopal gas leak [20,23,24]. As a result, it is necessary to detect any deterioration in OSH management systems and quantify the amount of accident risk and how it changes over time [5,8,25]. There are two indicators when reviewing safety management procedures: positive indicators that show potential for improvement and negative indicators that serve as early warning signs of management system failures. This proactive indication can help detect and manage safety issues before they turn into an incident or cause harm [5,8,16,26]. Proactive indicators can also be used as benchmarks for current practice to demonstrate continuous progress over time, monitor safety performance tolerance levels, and take action when these tolerance levels are breached [5,27,28]. However, reporting practices for occupational safety and health (OSH) vary by industry and workplace sector, depending on organizational structure, technology, and type of activity [16,29]. Further research is needed to establish more effective OSH performance indicators and assist businesses in implementing them [30].

Despite the rising relevance of examining proactive indicators, the literature on the utility industry is fragmented. Thus, this research aims to bridge the gap between identifying indicators used in assessing safety performance in the utility industries and their ties to safety outcomes to enhance safety in the utility business. This study aims to conduct a systematic literature review by grasping the concept of safety indicators, measuring techniques, identifying indicators of safety management practices used in utility industries, and the associations between indicators.

## 2. Methods

The methodology employed in this research was based on Preferred Reporting Items for Systematic Reviews and Meta-Analyses (PRISMA) to formulate the research question, systematic searching strategies, quality appraisal and data abstraction and analysis. The study was conducted from March 2021 till July 2021. This review included three main aspects in the review, namely the utility industry (population), indicators (interest) and safety management practices (context) [31] and aims to answer the following question: what types of indicators are used in safety management practices among utility industries?

### 2.1. Systematic Searching Strategies

**Figure 1:**
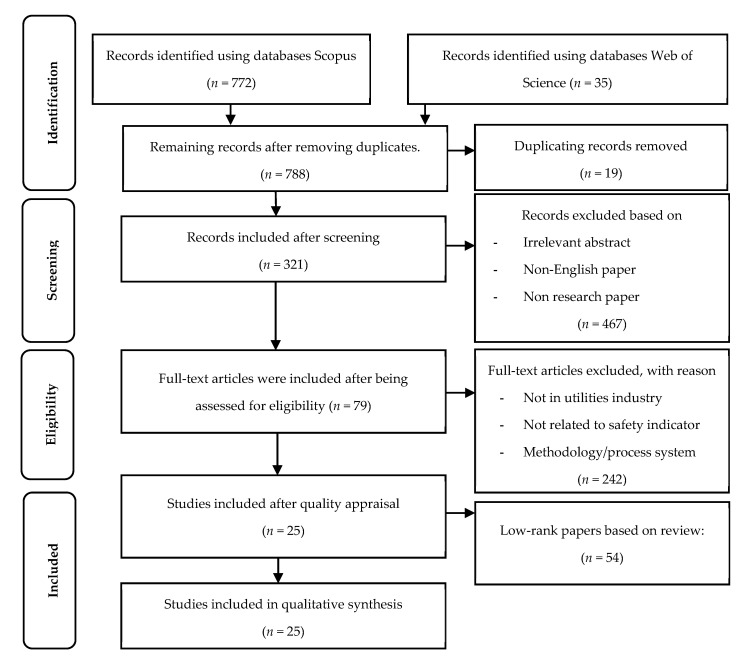
This describes the main processes based on PRISMA.

Three main processes in the systematic searching strategies process are the identification, screening, and eligibility based on PRISMA [32], as shown in Figure 1.

#### 2.1.1. Identification

Identification is a search process using the study’s main keywords, namely safety indicator, safety management practices, and utility industries which relied on keywords developed based on the research question [33]. To provide more options for selecting databases in the search for more related articles for the review, searching processes used any synonym, associated term, and variation. The identification process relied on past studies, keywords recommended by guidelines and keywords recommended by experts. Scopus and Web of Science databases were used in this research, using enriched existing keywords and developed full search strings, shown in Table 1. The searching process in these two databases was conducted from March 2021 to May 2021, with the published articles limited from 2000 to May 2021, resulting in 807 articles.

#### 2.1.2. Screening

This study screened all 807 selected articles by selecting the criteria for article selection, which was completed automatically using the database’s sorting function. The authors removed 19 articles that were duplicates from the selected articles. Furthermore, only articles with empirical data published in a journal were included in the review to ensure their quality. Additionally, only items written in English were included in the review to minimize misunderstandings. The inclusion and exclusion criteria shown in Table 2 were used to include 321 articles and exclude 467 articles to achieve the study’s objectives.

#### 2.1.3. Eligibility

Eligibility involved personally reviewing the retrieved articles to guarantee that all the remaining articles after the screening process met the research criteria. This procedure was accomplished by reading the title and abstract and skimming through the papers. The elimination process was based on unclear methodology, non-safety management practice indicators, conducted in non-utilities industries, not related to the safety and health field, and published as a chapter in a book. As a result, 242 articles were removed and 79 were chosen.

### 2.2. Quality Appraisal

Two specialists were chosen for quality appraisal with a background in safety and health and more than 15 years as an enforcer and auditor of safety management system certification. The remaining articles were sent to the specialists for assessment to ensure that the content was high quality. The remaining articles were categorized into three categories: high, medium, and low, with high and moderate papers being reviewed [34,35]. The articles were categorized when both specialists agreed with the ranking decision. When there was disagreement between the categories addressed, the lowest rank given by either one of the specialists was chosen. This approach yielded 9 high-ranking articles, 16 moderate-ranking articles, and 54 low-ranking articles. As a result, articles with a low ranking were eliminated, leaving only 25 articles suitable for examination.

### 2.3. Data Abstraction and Analysis

This research study chose the qualitative strategy to synthesize or analyse integrative data [36]. The researcher read the full text for all 25 articles, focusing on the abstract, findings, and discussion sections. Data abstraction was carried out based on the research questions, meaning any data from the evaluated study that can answer the research question and were then entered into a table. Thematic analysis was then used to identify indicators and sub-indicators within the abstracted data based on noticing patterns and themes, clustering, counting, noting similarities, and relationships [37].

The first stage in thematic analysis is to produce indicators by looking for patterns in the abstracted data in all the articles reviewed for similarity. Based on a comparison of the conceptual theory of indicators for similarity, the comparable and abstracted data were pooled into three main indicators. The three sets of data were further analysed and synthesized, revealing another 15 sub-indicators. The data were divided into three main indicators: safety management practices acting as a driven leading indicator, safety performance behaviour acting as an observer leading indicator, and safety outcomes acting as a lagging indicator. There were seven sub-indicators in the safety management practices group, four in the safety performance group, and four in the safety results group.

## 3. Results

### 3.1. Temporal and Spatial Distribution

The review consisted of identification, screening, eligibility, and included processes, thus, obtaining 25 selected articles related to the research question. The review’s main indicators were safety management practices, safety performance behaviour, and safety outcomes, and resulted in 15 sub-indicators, as shown in Table 3. Then, seven sub-indicators under safety management practices that act as driven leading indicators were identified: management commitment, involvement of workers, hazard identification and assessment, hazard prevention and control, training and education, evaluation and improvement, and communication and coordination. Meanwhile, the indicators for safety performance behaviour acting as an observant leading indicator consisted of four sub-indicators: safety motivation, safety knowledge, safety compliance, and safety participation. Lastly, the safety outcomes indicators that served as lagging indicators were identified, consisting of four sub-indicators: occupational accidents, occupational fatal accidents, near misses, and lost time injuries.

**Figure 2:**
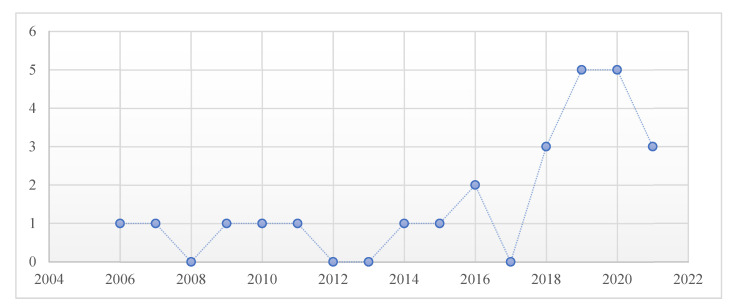
Number of reviewed papers selected by year published.

According to Figure 2, the maximum number of articles on safety management practice indicators in the utility industry was published in 2019 and 2020, with five articles (20%) each year. The distribution of publications fluctuated during the decade, with only one article published each year from 2006 to 2015 and then increasing in 2016. However, due to the review research being conducted in May 2021, the number of publications released in 2021 appears to be declining. It is expected that more articles will be published throughout the rest of the year. No papers were published from 2000 until 2005, then none in 2008, 2012, 2013, and 2017. The fluctuation trends in the number of published articles showed that researchers focused on proactive actions to anticipate safety issues and potentially reduce OSH incidents.

**Figure 3:**
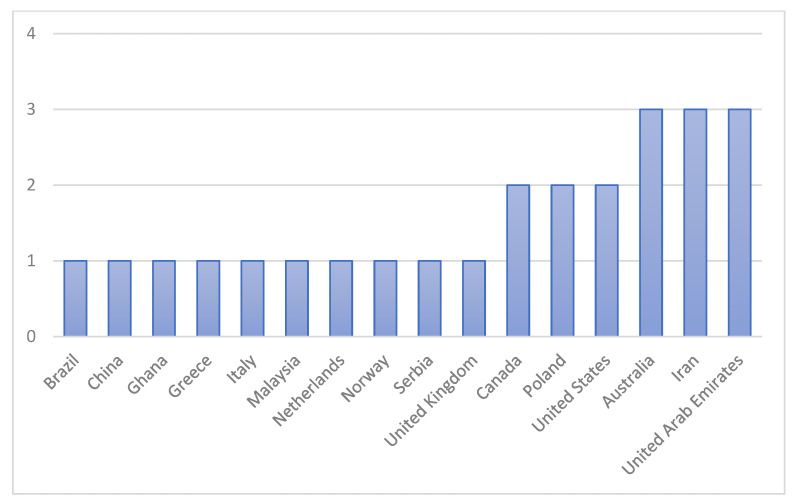
Number of reviewed papers selected by country.

Figure 3 shows the number of articles according to their country of origin. Most of the studies were conducted in Australia with three articles (12%), Iran with three articles (12%), and the United Arab Emirates (UAE) with three articles (12%), followed by Canada with two articles (8%), Poland with two articles (8%), and the United States (US) with two articles (8%). Most countries only published one article: Brazil, China, Ghana, Greece, Italy, Malaysia, Netherlands, Norway, Serbia, and the United Kingdom (UK).

The review articles published were focused on general sectors with 10 papers (40%), followed by the gas utility sector with 9 papers (36%), and the electricity utility sector with 6 papers (24%). In their sampling, the articles that researched multiple or various industries, including the utility sectors, were included in this systematic review and were known as the general sector due to their suitability for the utility industry’s safety management practices. From the 25 articles selected, most of the studies were conducted on driven leading indicators with 22 papers (85%), followed by observant leading indicators with 10 papers (38%), and lagging indicators with 8 papers (31%).

Leading indicators can be measured as passive, objective, or subjective. Most of the studies focused on subjective measurement with 20 articles (77%), followed by objective measurement with 6 articles (23%), and passive measurement with 2 articles (8%), from the 25 articles selected. Leading indicators research was distinguished into two phases: the development phase, which included defining, developing, or measuring, and the progressive phase through validation testing of leading on lagging indicators. Based on the selected articles, most of the studies were in the development phase with 18 articles (72%) and the progressive phase with 7 articles (28%).

### 3.2. Driven Leading Indicators

These studies on safety management practices were a group as the driven leading indicators. They were assessed through seven indicators: management commitment, workers’ involvement, hazard identification and assessment, hazard prevention and control, training and education, evaluation and improvement, and communication and coordination.

Management commitment is an internal safety factor that relates to how senior management appears to prioritize safety issues, communicates well, and acts effectively in an organization that values safety [47]. Thematic analysis conducted in this research shows that 22 articles (85%) studied management commitment. Indicators for successful implementation of safety management systems depend on top management to develop safety policies; OSH leadership, visible commitment, and safety as core values can shape the safety climate and performance to influence positive and lasting effects on safety.

Workers’ involvement in safety can improve safety performance in an organization, as workers are the best-qualified people to make improvement suggestions because they are the people closest to the job. The thematic analysis found that 15 articles (58%) studied workers’ involvement in safety management practices. Workers’ involvement can be measured through the level of involvement encouragement, empowerment for safety, worker consultation, and removing barriers for workers’ involvement that will lead to their ‘ownership’ towards safety.

The analysis found that only 11 articles (42%) discussed hazard identification and assessment practices in safety management. Hazard identification and assessment are important in identifying and verifying hazards to support the efficient functioning of safety management systems. Through this practice, the prevention of accidents or similar undesirable events from reoccurring can be achieved. This practice is measured through four indicators: identifying existing hazards, workplace inspections, accident investigation, and hazard assessment.

Hazard prevention and control are essential in ensuring adequate hazard controls are implemented and operated effectively. Thematic analysis shows that 14 articles (54%) studied hazard prevention and control. There are four indicators used in assessing hazard prevention and control practices: planning, implementing, managing, and verifying hazard controls. This practice can lead to proactively improving, ensuring implementation, continuous implementation, and verifying control effectiveness.

Training and education were the second-most-studied factor in the review, consisting of 18 articles (69%) out of 25 papers. This practice can be enhanced through management commitment towards safety training that leads workers to gain knowledge, awareness, and ability to recognize hazards, thus, increasing safety levels. Thus, training and education are measured through four indicators: management roles in training, the effectiveness of workers’ training, training on hazard identification and control, and safety awareness.

The safety management systems require an evaluation for the implementation and corrective actions of documented and implemented measures. This practice was studied in 16 articles (62%) from the selected review papers, studying performance evaluation of safety programmes, safety audits, identification of weaknesses, and identification of opportunities. It is important to keep track of performance appraisals and audits, which are essential to detect and describe safety programmes and management conditions. Weakness identification is important to avoid adverse safety incidents following unsuccessful work operations; thus, continuous improvement can be implemented by controlling and reviewing activities, so that performance goals and indicators remain relevant.

Communication and coordination help organizations manage safety issues and progress related issues between organizations with diverse objectives from potential hazards and accidents. This practice is studied in 13 selected articles (50%) and can be measured through four indicators: management communication, safety reporting, supervisory communication, and OSH coordination. Effective safety communication and coordination between managers and workers are important to communicate safety problems or concerns that lead to a positive safety climate. Further, proactive supervisors will emphasize supervisory monitoring practices by being committed to safety, thus, ensuring workers and contractors follow safety rules. These safety management practices and their indicators are detailed in Table 4.

### 3.3. Observant Leading Indicator

One method that can be used to observe the effectiveness of programmes or activities is by measuring employee safety behaviours. In this systematic literature review, the author identified two main indicators in observant leading indicators: proximal safety antecedents and safety behaviours. Proximal safety antecedents consist of safety knowledge and safety motivation, and safety performance consists of safety compliance and safety participation. Most of the studies focused on safety compliance with 11 articles (42%), followed by safety participation with 8 articles (31%), safety motivation with 5 articles (19%), and safety knowledge with 3 articles (12%).

Safety knowledge is the awareness of proper methods for performing safe behaviours as proximal antecedents of safety performance or mediators of the relationship between personality traits or job and related organizational factors and safety performance [45,59,60]. Safety knowledge is measured through a scale of six items, namely workers knowing how to perform the job safely, how to use safety equipment and standard work procedures, how to maintain or improve safety and health in the workplace, how to reduce the risk of accidents and incidents in the workplace, the associated hazards and necessary precautions, and reporting potential hazards noticed in the workplace [45]. Another proximal antecedent of safety performance was safety motivation, which refers to the enthusiasm to implement safety behaviours and the courage associated with those behaviours [45,60]. Safety motivation is measured through a scale of three items: efforts to maintain or improve personal safety, the importance of maintaining safety at all times, and the importance of reducing the risk of accidents and incidents in the workplace [54]. In meta-analysis studies, the safety climate was positively related to safety knowledge and safety motivation, both being related to predicting safety performance, which indirectly influences accidents and injuries [60]. Workers’ health and safety can be improved through investment in knowledge and training that encourage safe behaviour [59,61].

Safety performance has been conceptualized as individual behaviours with a measurable criterion proximally related to psychological factors more than accidents or injuries that can be distinguished into safety compliance and participation [60]. Safety compliance refers to workers’ behaviour in following safety policies and procedures towards meeting work safety standards, such as complying with personal protective equipment requirements, carrying out tasks safely, obeying safety regulations, and using correct procedures [53]. On the other hand, safety participation refers to workers’ behaviour in helping create an atmosphere supportive of safety that moves beyond procedures to assist colleagues, engage in voluntary safety activities, promote safety and its principles, take safety initiatives, and improve workplace safety [53]. Safety practices and leading indicators have positive and strong associations with safety compliance and safety participation [45,48,54].

### 3.4. Lagging Indicators

The authors identified the lagging indicators that represent the safety outcomes based on the review papers: occupational accidents, occupational fatality accidents, near misses, and lost time injuries. Most of the lagging indicators studied were occupational accidents in eight articles (31%), followed by lost time injuries in five articles (19%), occupational fatal accidents in four articles (15%), and near misses in four articles (15%).

Occupational accidents are referred to as accidents that result in injuries needing medical attention [60]. The reduction in occupational accidents is considered the final goal or outcome of safety efforts in an organization [52]. Occupational accidents are the outcomes of many factors, including unsafe behaviour, which was a direct trigger factor, with injuries representing low-base-rate and count variables [59] in most organizational measured injury rates [51]. Occupational accidents can also be measured by recordable injuries resulting in lost time, recordable injuries requiring medical treatment, and incident rates based on severity and frequency [44,55]. It was found that only five papers discussed or mentioned lost time injuries as a lagging indicator. Fatality was mentioned as the second type of severity related to high-consequence injury and illness resulting in death [44,51,55].

Another lagging indicator is measured through lost time injury. There are two ways of reporting lost time: lost time injuries, which refer to the subset of work-related injuries that result in ‘lost time’ due to work absence, and lost time injury frequency rate, which is defined as the number of lost time work-related injuries (fatalities and lost workday cases) per 1,000,000 work hours [40,55]. However, some firms calculated lost time injury frequency rates based on U.S. Occupational Health and Safety Administration Guidance, which uses 200,000 h as the denominator [55]. Prior research labelled lost time injury as a lagging indicator [39,40,44,51,55].

Near misses are lagging indicators resulting from inadequate safety efforts and are defined as unplanned incidents with short-term results that do not result in an accident or injury [52,59]. However, research shows that workers tend to under-report near misses, causing the relationship between these variables and their predictors to be attenuated [59]. Near misses can also be considered a leading indicator, measured by the number of near misses investigated [44].

## 4. Discussion

### 4.1. Current Practices and Progress

The number of published articles regarding indicators used in safety management practices in the utility industries has increased in recent years, from 2000 until 2021. The increasing number of published papers show that there has been a high awareness that safety lagging indicators, such as injury rates, have limited use in preventing future injuries. Thus, proactive measures through predictive measurements can provide early warnings of potential hazards to improve future performance [7,8,65]. For this reason, there is a need to proactively measure and identify the adequacy of safety management practices at an early stage to predict any deterioration in safety management system implementation, thus, contributing to positive safety outcomes.

Most papers were published in the United Arab Emirates, Iran, and Australia. The United Arab Emirates and Iran published papers focused on the gas utility industry, the primary players in the oil and gas industries [47,66]. As a leading country in the oil and gas industry, it is essential to ensure supply and productivity are guaranteed in occupational safety and health to avoid disasters or accidents that will disrupt the production process. Thus, it is vital to ensure the effectiveness of a safety and health management system that can eliminate injuries, adverse health impacts, and damage at the operational level, thus, improving the productivity of workers and their physical and mental well-being and workplace satisfaction [40], thus, showing the importance of proactive indicators in reducing unwanted events in the workplace through the implementation of safety management practices as proactive efforts.

The majority of the selected articles were studied to identify and develop driven leading indicators. These indicators are essential in assessing and improving the functioning of sociotechnical systems as part of an organizational safety management process [17] that contains safety antecedents as input into safety efforts and measures of any actions to produce the output that can directly or indirectly influence safety performance [52,60]. Leading indicators contain input and activity elements that are critical for safety decisions in the organization to achieve safety objectives [18,19,30,52]. Thus, safety management practices are considered the antecedent of the safety climate for organizations to improve safety performance. The extensive distribution of studies in the systematic literature review of safety management systems among utility industries indicates that the development phase of leading indicators is very encouraging. This phase involves the identification, development, and measurement of leading indicators. Thus, leading indicators are well defined in ensuring that safety management systems are maintained comprehensively through activities conducted in an organization.

Another finding was that most of the reviewed papers studied subjective indicators in measuring leading indicators. The subjective data are often obtained through surveys or questionnaires with advantages in collecting relative measurements and perceptions, such as quality. The main drawback is that these indicators are difficult and expensive to manage, even when datasets are obtained, monitored, and maintained in the same way as organizations maintain objective performance data [18,67]. Nevertheless, subjective measurement was often used in measuring the level of safety in an organization in the research [68,69,70,71,72,73]. Subjective measurement is based on perceptions towards activities implemented in studying a programme’s effectiveness in reaching workers as a target group in organizations. It shows that subjective measurement through a perception measurement scale is the appropriate method for collecting proactive indicators that measure the quality of activity implementation.

Management commitment is an internal factor in an organization, related to self-regulation that significantly influences the safety behaviour of workers and is essential for the success of safety management systems [48,74,75]. This study found that safety management practices focused on management commitment practices as the leading indicators for measuring safety levels in organizations, showing that an effective safety management system relies on top management developing company policies and setting resources. It supported stable, consistent, and fair OSH leadership in management commitment, which impacted OSH management system effectiveness to the greatest extent [49,76]. Low accident rates are also associated with administration, showing inspirational motivation by fostering safety goals, promoting safety, and motivating workers to engage in safety behaviours [74,77,78,79,80]. The study found that only five articles discuss transformational leadership. Transformational leaders are the key element to high-safety performance that influences worker’s safety behaviours and safety climate [38,48,53,59,60]. Thus, management leadership plays an essential role in influencing employee safety performance through safety involvement. In turn, improving safety behaviours will reduce accidents, injuries, and absenteeism. Top management has the final say in decision making, as consultation with workers is only supplementary in getting more information towards making the final decision. Authentic OSH leadership always puts safety as a priority and core value in organizations to ensure the safety of workers in the workplace.

Observant leading indicators are another leading indicator related to safety management practices through thematic analysis. These indicators are defined as indicators that provide insights into dynamic systems in the form of questions regarding the activities taking place, the capabilities, skills, and motivations of personnel, routines, and practices, as well as the potential of the organization for safety [17], in which individual behaviour is an important performance metric to measure and observe the effectiveness of safety activities implemented by organizations [52,74,81]. Most review papers found that safety management practices positively predict safety compliance and safety participation, showing observant leading indicators are important indicators in reducing occupational injuries and accidents.

Lagging indicators are the results of activities or events that aim to reduce accidents and injuries through safety efforts within the organization [17,52]. Safety outcomes are measurable and clear to the organization and they include negative performance indicators, such as the number or frequency of accidents at work, the cost of compensation to workers, the number of days not worked due to occupational accidents, and the number of occupational diseases [30,60]. The most studied lagging indicators in the selected papers were occupational accidents, followed by occupational accident fatalities, lost time injuries, and near misses. Accidents result from numerous factors, and individual unsafe behaviour is one of the most direct trigger factors. The severity of an accident is measured by its effect on injuries and property damage. Briefly, an incident analysis will show something about accidents, such as weaknesses in OSH programmes and activities.

The studies found that researchers from the selected papers focus on practical research. It has been shown that seven leading indicators in driven leading indicators were based on a standard, such as ANSI Z10, ISO 45001, and Occupational Safety and Health Administrator guidelines. The safety outcomes proposed by researchers are also in line with the standards and regulations in a particular country, which have also been used by ILO in capturing data regarding occupational safety and health issues, thus, showing that current research is based on practical and industrial-oriented factors.

### 4.2. Limitations and Challenges

Based on the current thematic analysis results, the number of selected review papers on safety management practices in the utility business is still modest and has only increased in recent years. Most papers are published in the gas utility field rather than other utility industries, such as water utilities, electrical or power utilities, and sanitary services. Additionally, most of the papers were removed in the screening process due to the research’s focus mainly on the construction industry, thus, indicating a gap in the research implemented in these industries that needs to be investigated. Since OSH reporting procedures vary by industry and workplace, additional research is required to identify OSH performance indicators that are more auspicious and can assist firms in implementing them [16,30]. Future research is needed in a broader range of utility industries, which may have more informal OSH standards and procedures by adapting or benchmarking tools across different safety management activities.

Compared to subjective measurements, passive and objective indicators were less studied in measuring safety management practices. Passive indicators designate the likelihood of safety performance being achieved, usually through binary feedback, instead of objective indicators that measure the frequency and subjective indicators that measure the quality of execution that may change over time [26,82]. The main reason objective indicators were less studied could be that the quality of existing systems or activities may not be measurable through objective measurements. Furthermore, objective indicators are likely to be manipulated and distorted to improve the appearance of the organization [18]. However, future research should measure both objective indicators for key performance indicators of activities implemented and subjective indicators for the quality of the activities. Along with that, indicator selection should be based on specific, measurable, accountable, reasonable, and timely criteria.

Practices for hazard identification and assessment were under-represented in the publications selected for this study. This practice is an initial step in risk management to identify the causes and mechanisms of undesirable events by assessing the likelihood of the event and the severity caused by the event. Therefore, systematic planning in eliminating or reducing safety hazards is essential in safety management to improve the safety climate [83,84,85], depending on proactive, ongoing processes and an assessment of hazard elements [86]. Inadequate hazard identification is one of the key contributing causes of fatal workplace accidents, affecting corporate values, such as ethics and profit. Hazard should be identified and controlled before work is carried out to ensure safety issues are under control but usually take a long time to be eliminated or controlled, thus, increasing the probability of accidents [87,88]. Accordingly, it is crucial to integrate risk management practices into the safety management system to increase the effectiveness of implementing this system in reducing accidents. Integrating risk management practices into safety management will also support the implementation and certification of current safety management systems, such as ISO 45001:2018, that emphasise preventive measures. However, this study has limitations in assessing hazard identification and control findings as it was an understudy in the selected papers. Mainly, risk management was studied in stand-alone research that separated from the safety management system. Future research should incorporate risk management as a crucial indicator to be monitored, essential in preventing occupational accidents and diseases.

Based on the research conducted, occupational accidents resulting in injuries received more interest in the selected papers. They were supported by Tsalis et al. [51], who found that most organizations provided more information about injury rates. However, attention should be given to all types of accidents, regardless of the degree of damage or loss, such as fatalities, occupational accidents, lost time, and near misses. Accidents that do not result in injury or damage to equipment and materials still need attention as they are signs of future accidents. Further, near miss reporting should be considered a lagging indicator, since luck is often the only difference between a near miss and a fatality [89]. Future studies should incorporate occupational accidents, fatal occupational accidents, lost time injuries, and near misses as safety outcomes. However, researchers should also focus on positive outcomes, such as productivity, monetary increase, and profit increase due to high safety levels in organizations. Thus, it will encourage management to further implement safety and health in the workplace.

This study found an inadequate correlation between driven leading indicators, observant leading indicators, and lagging indicators. It can be seen through the progress of research, which shows that studies focused on developing indicators that included defining, developing, and measuring the indicators. On the contrary, the analysis focused on progressing the indicators that study the relationship between driven leading indicators and observant leading indicators or when lagging indicators are small in number. In meta-analysis research, proactive measurement through situations and individual difference factors, such as safety antecedents, were negatively related to safety outcomes through proximal antecedents and safety performance [60]. However, Jiang et al. (2010) found a lack of evidence on safety management practices as predictors and near misses’ relationships due to the probability of underreporting [59]. Thus, the correlation between leading indicators and safety outcomes is complicated [90]. Future studies should focus on validating the influence of leading indicators in safety management practices toward safety outcomes. Researchers should try to correlate leading indicators and lagging indicators (safety outcome) to better understand the implementation safety management systems in reducing safety and health issues at the workplace, thus, ensuring productivity is sustained.

Other limitations in this research were due to the focus of the study on leading indicators, mainly in the construction industry compared to the utility industry, thus, making the papers selected for review smaller in number. The study also found that research on leading indicators is limited in quality. Most was published in lower-rank journals with restricted access, making it challenging to review and choose these as selected papers. Most of the documents on leading and lagging indicators were mainly on the concept and theoretical aspects, lacking evidence in empirical analysis.

## 5. Conclusions

The present study reviewed 25 articles on indicators used in safety management practices in the utility industries, reflecting an understanding of current practices and progress. This study also revealed the potential use and the gaps in knowledge on the use of indicators in safety management practices, plus several subject areas that can be researched further. It was found that the number of studies on proactive measurement in the utility industries has increased in recent years. Most of the studies were conducted in the United Arab Emirates, Iran, and Australia. Furthermore, three main indicators that represented the use of indicators in safety management practices among utility industries were identified based on the systematic review performed. The most researched indicators were driven leading indicators, which were described as indicators that assess and improve the functioning of sociotechnical systems as part of organizational safety management. There was an imbalance in terms of the type of area researched for sectors in the utility industry. Most of the studies focused on gas and electrical utilities compared with water utilities and sanitary services. Furthermore, most of the research focused on management commitment as an essential element in safety management practices, thus, creating an imbalance in practices. The majority of the study focused on identifying, developing, and measuring leading indicators. These findings indicate plenty of opportunities for discovery and new research for OSH practitioners, authorities, and researchers to explore, in terms of the use of indicators to enhance safety management practices in the utility industry.

This systematic review paper confirms several limitations and gaps in the study of indicators used in safety management practices in the utility industries in recent years. Firstly, information on indicators used in safety management practices from other countries and sub-industries among utility industries is still lacking. Future research is needed in a broader range of utility industries, which may have more informal OSH standards and procedures by adapting or benchmarking indicators across different safety management practices. Further, there is still a lack of information on objective data measuring implementation instead of subjective data measuring perception. Thus, in future research, researchers may measure both objective indicators for key performance indicators of activities implemented and subjective indicators for the quality of the activities, which can change from time to time. Inadequate hazard identification and assessment practices in the utility industries were also reported in this study. As a result, researchers should incorporate risk management strategies into safety management systems in future research. Occupational accidents that emphasize injury were the most reported lagging indicators used as safety outcomes in this research. Thus, there is a need to include occupational accidents, fatalities, lost time injuries, and near misses as safety outcomes in future studies. Finally, the development phase of research, which includes identifying, developing, and measuring indicators, was dominant compared to the progressive phase on the indicator used in safety management practices. Next, it is recommended for future studies that researchers explore the relationship between driven leading indicators and observant leading indicators towards lagging indicators. Therefore, further broadening this basic understanding through the integration of diverse research findings may assist the concerned parties in enhancing safety levels, such as OSH practitioners, authorities, and researchers, in developing strategies that align with the needs, abilities, and interests of safety.

## Figures and Tables

**Table 1:** The search strings.

Database	Search String
Web of Science	TOPIC: (“safety indicators” OR “key safety performance indicators” OR “safety performance indicators” OR “safety performance outcomes” OR “safety performance” OR “OHS performance” OR “safety outcome indicators” OR “leading indicator” OR “lagging indicator”) Refined by: TOPIC: (“safety management systems” OR “safety management practices” OR “safety system practices” OR “safety management programs” OR “safety programs” OR “risk management” OR “safety measures”) AND TOPIC: (utilities OR water OR electrical OR electricity OR “electrical supply” OR “power supply” OR “power transmission” OR “electric transmission” OR gas OR “sanitary services”)
Scopus	(TITLE-ABS-KEY (“safety indicators” OR “key safety performance indicators” OR “safety performance indicators” OR “safety performance outcomes” OR “safety performance” OR “OHS performance” OR “safety outcome indicators” OR “leading indicator” OR “lagging indicator”)) AND ((“safety management systems” OR “safety management practices” OR “safety system practices” OR “safety management programs” OR “safety programs” OR “risk management” OR “safety measures”)) AND (utilities OR water OR electrical OR electricity OR “electrical supply” OR “power supply” OR “power transmission” OR “electric transmission” OR gas OR “sanitary services”))

**Table 2:** The inclusion and exclusion criteria.

Criteria	Inclusion	Exclusion
Publication timeline	2000–May 2021	1999 and before
Document type	Article (with empirical data) and review	Conference proceedings, chapters in book, book series, books, etc.
Language	English	Non-English
Nature of the study	Measurement of current safety level Safety management practices in industries Safety outcomes	Research of method/process system Not related to safety indicators Not related to utilities industries

**Table 3:** The groups and sub-groups.

[Reference] Author (Year), Country	Driven Leading Indicators (Safety Management Practices)							Observant Leading Indicators (Safety Behaviour)				Lagging Indicators (Safety Outcomes)			
	MC	WI	HI	HC	TE	EI	CC	SK	SM	SC	SP	OA	FA	NM	LT
[38] Barker (2021), Canada	X	X		X	X	X	X		X	X					
[39] Zarei et al. (2021), Iran	X	X	X	X	X		X								
[40] Sarkheil (2021), Iran	X				X	X						X			X
[41] Zwetsloot et al. (2020), Netherlands	X	X	X	X	X	X	X								
[42] Al Mazrouei et al. (2020), UAE	X	X			X	X	X								
[43] Janackovic et al. (2020), Serbia	X		X	X	X	X	X								
[44] Ahmed Naji et al. (2020), Malaysia	X	X		X	X	X					X	X	X	X	X
[45] Rajabi et al. (2020), Iran	X	X			X	X		X	X	X	X				
[46] Al Mazrouei et al. (2019a), UAE	X	X				X	X								
[47] Al Mazrouei et al. (2019b), UAE	X									X					
[48] Casey et al. (2019), Australia	X	X	X	X	X	X	X			X	X	X		X	
[49] Skład (2019), Poland	X					X				X	X				
[50] Santos et al. (2019), Brazil	X	X	X	X	X	X	X								
[51] Tsalis et al. (2018), Greece		X		X	X							X	X	X	X
[52] Mousavi et al. (2018), Italy	X	X	X	X	X	X	X			X	X	X	X	X	
[53] Dartey-Baah & Addo (2018), Ghana	X									X	X				
[54] Shea et al. (2016), Australia	X	X	X	X	X	X	X		X	X	X				
[55] O’Neill et al. (2016), Australia												X	X		X
[56] Podgórski (2015), Poland	X	X	X	X	X	X	X								
[57] Becker (2014), Canada						X									
[58] Øien et al. (2011), Norway	X		X		X	X									
[59] Jiang et al. (2010), China	X	X		X	X			X	X	X	X	X		X	
[60] Christian et al. (2009), US	X		X	X			X	X	X	X	X	X			
[61] Yule et al. (2007), UK	X	X			X					X				X	
[62] Liggett (2006), US	X		X	X	X		X								
**Safety Management Practices**	**Safety Performance Behaviour**								**Safety Outcomes**						
MC = Management Commitment	SK = Safety Knowledge								OA = Occupational Accident						
WI = Workers Involvement	SM = Safety Motivation								FA = Occupational Fatality Accident						
HI = Hazard Identification & Assessment	SC = Safety Compliance								NM = Near Misses						
HC = Hazard Prevention & Control	SP = Safety Participation								LT = Lost Time Injury						
TE = Training & Education															
EI = Evaluation & Improvement															
CC = Communication & Coordination															

**Table 4:** Safety management practices leading indicators extracted from reviewed articles.

Aspect	Leading Indicators	References
**Management Commitment**		
Safety policy	A clear safety vision and objectives; Implementated by managers and workgroups; Workers’ knowledge and awareness on safety policy; Provision to establish procedures and control; and The number of policy reviews and updates.	[38,41,46,49,52]
Management leadership	Inspiring and motivating through words and actions; Gaining trust through charisma and being exemplary; Having committed and competent management; and OSH issues are in top management meeting agendas	[38,41,46,49,52]
Visible management	Active engagement and promotion; Providing assistance and support for improvement; Implementing workers suggestions; Identifying and monitoring worker’s deviations and errors; Informal interactions inside and outside the workplace; Emphasis on safety procedures and policies; Setting individual and company safety goals; Regular two-way communication; Safety walkthroughs by top managers; and Rating of management commitment in OSH management.	[41,43,45,46,48,52,53,55,60]
Core values	Provision of adequate funds and resources; Procedures, training programmes, and competence selection; High priority for safety; and Budget spent on OSH improvement activities.	[39,42,43,48,53,55]
**Workers’ Involvement**		
Encouraging involvement	Leader engagement with workers; Workers’ understanding and commitment on values and goals; Sufficient budget allocation; Workers’ are recognized, valued, and rewarded; Open-door policy; Management take serious on OSH issues and suggestions; Having effective OSH committees; and Meetings commissioned on OSH issues.	[38,41,42,44,46,48,51,55,58,62]
Empowerment for safety	Active participation in safety decision making; Shared responsibility and accountability with workers in making safety decision; Workers participate proactively in safety efforts and monitoring of the workplace; OSH improvements proposed by workers or their representatives; and Risk assessment activities conducted with workers’ involvement.	[38,39,48,53,55]
Worker consultation	Workers’ perceptions towards OSH; Consulting on safety issues directly with workers; Collaboration and shared planning; Seeking information from workers; Support to ensure task objectives is achieved; Consultation in developing procedures; and Allowing workers to make suggestions for the improvement.	[45,48,53,58]
Removing barriers for involvement	Improving policy regarding workers’ participation in safety; Equal status distinctions to all workers in giving input and information on safety; Providing timely feedback; Rating effectiveness involvement; and Allocation on OSH incentives and budget.	[38,45,48,55,58,62]
**Hazard Identification and Assessment**		
Identifying existing hazards	Addressing workers’ to all hazards associated with the workplace; Workers’ understanding on hazards and how to protect themselves; Integrating OSH in pre-work briefings on identified specific hazards and risks; Assessing hazards through job safety analysis; Consideration of ergonomic factors, reviewing designs, standards and regulations; and Identifying any risks before internal changes are made.	[41,55,61,62]
Workplace inspections	Identifying hazards associated with work and production pressures which influence safety performance; Identifying hazards associated with psychosocial, physical or physiological factors; Verifying regular maintenance of all equipment; and Ensuring hazards are controlled and equipments are installed correctly and safe.	[53,59,61]
Accident investigation	Identified hazard through reports of accidents and safety issues; Identifying root causes of the incident; Evaluating the quality of the frameworks, procedures, or interventions implemented; Adequate follow-up of reported unplanned events; Increase in the reporting rate; The quality of incident investigation and analysis; How lessons learned are communicated; and Measuring the ratio between accidents that occurred and near misses reported.	[41,44,50,59]
Hazard assessment	Integrating risk management in the OSH management that includes risk assessments; Workers’ involvement in hazard assessments; Helping workers to perceive the risks associated with the job, the accident potential, physical hazards, and job safety; Assessing safety levels on human, organizational and environmental indicators; and informing workers of the results of risk assessments due to changes introduced.	[43,48,53,55,59]
**Hazard prevention and control**		
Planning hazard controls	Proactively improving OSH from the design phase; Integrating risk and OSH management; Response to human performance problems; and Planning for the job and task.	[41,53,57,61]
Implementing hazard controls	Selective hiring based on fitness for the job; Implementing working procedures or interventions; Executing temporary control; Timely corrective actions, maintenance and checking false reports; and Numbers of controls implemented based on hierarchy.	[38,43,44,48,53,55,58,59]
Managing hazard controls	The awareness of employees of current risk levels, controls, and conditions; Written OSH procedures and safe working; Assessing behaviour and human error; Equipment maintenance to safe standards; and The number of safety grievances addressed and resolved.	[39,43,48,51,55,58,61,62]
Verifying hazard controls	Enforcing non-compliance; standardization of work procedures; Supplying workers with personal protective equipment, correct tools and equipment, using precisely installed equipment; and Reviewing and evaluating corrective actions.	[48,50,55,61,62]
**Training and education**		
Management roles in training	Training provisions that inspire positive attitudes and an energetic environment; Time allocation and planning for safety training; providing adequate safety training; Maintaining training records; Investing in workers’ training and knowledge; Managers participated in OSH courses; and Workers are trained on their duties and responsibilities.	[42,43,44,46,50,51,53,55,60]
Effectiveness of workers’ training	The numbers of workers trained; Safety induction for new recruits and contractors; Improvement in qualifications through skills, competency, and knowledge; Continuous development with regular and refresher training; and Workers are trained for critical positions and qualified before commencing work.	[41,43,44,46,50,55,58,63]
Training on hazard identification and control	The ability of workers to assess hazards and control measures in the workplace; Workers familiarization with procedures, standards, practices, and equipment; Adequate training for responses and anticipation to a variety of threats or emergencies; and Safety skills across multiple domains.	[39,42,46,48,58,60]
Safety awareness	Workers level of awareness of hazards; Workers’ participation in safety OSH courses; Workers attitudes towards safety; Safety performance enhancement; and Workers awareness on their duties and responsibilities.	[38,42,45,46,55,61]
**Evaluation and improvement**		
Performance evaluation of safety programmes	The effectiveness of management targeted processes and programmes on safety goals; Safety standards compliance performance; OSH improvement goals in delivering results; Budget spent on plans, quality and effectiveness of OSH improvement; and Safety data collection.	[38,41,43,44,48,55]
Safety audits	Structured process in gathering information on pre-determined protocols; Evaluate OSH programs and management systems; Validating workers competency to ensure the sustainability of preventative and control measures; Compliance on OSH regulations and standards; and Audit conducted by external, experienced and assertive auditors.	[38,43,49,50,55,56]
Identification of weaknesses	Investigations to uncover causes of incidents and near misses; Investigations into nonconformities for corrective actions; Completion of corrective measures in due time; and Statistical reviews of occupational injuries.	[42,43,44,49,57,64]
Identification of opportunities	Evaluating high-quality work to improve job security and role overload; Measuring the effectiveness and sustainability of OSH promotions and sharing lessons learned; Periodically reviewed and improved operational procedures and OSH instructions; Positive feedback and recognition for past performance given; Nonconformities investigated for the potential for improvement; and Assessments made for technological solutions available.	[38,41,43,45,48,49,53,55]
**Communication and coordination**		
Management communication	Regular communication and interaction on safety goals; Sharing safety information by two-way and open discussions; Information flow and dissemination on work management and actual practices; Quantification of the communicational capacity of workers; Communication through verbal instruction, brochures, emails, or bulletins; Communication and consultation through formal and informal; and External OSH informational materials distributed internally.	[38,39,43,46,48,53,55,62]
Safety reporting	Applying scrutiny and transparency in reporting; Protection for workers reporting OSH issues or problems; The number of external OSH reports; Sharing information on accidents or near misses; and Communicating workers’ ideas and views on solutions for improving safety.	[49,50,53,55,61]
Supervisory communication	Regular interactions and guidance; Supervisors trained on hazards; and Supervisors valuing safety as reflected in communication, encouragement, and consequences.	[42,46,59,61]
OSH coordination	Pre-planning, planning and organization of work; Evalution of OSH risks during procurement; Managing contractor; monitoring contractor safety performance; and The quality of communication between the workgroup and stakeholders.	[41,44,50,59,62]

## Data Availability

Not applicable.
